# Grisel's Syndrome in Children: Two Case Reports and Systematic Review of the Literature

**DOI:** 10.1155/2020/8819758

**Published:** 2020-11-12

**Authors:** Nicole Pini, Martina Ceccoli, Patrizia Bergonzini, Lorenzo Iughetti

**Affiliations:** ^1^Post-Graduated School of Pediatrics, Department of Medical and Surgical Sciences for Mother Children and Adults, University of Modena and Reggio Emilia, Via Del Pozzo 71, Modena 41124, Italy; ^2^Pediatrics Unit, Department of Medical and Surgical Sciences for Mother Children and Adults, University of Modena and Reggio Emilia, Via Del Pozzo 71, Modena 41124, Italy

## Abstract

*Background and Objective*. Grisel's syndrome is a rare syndrome characterized by nontraumatic rotatory subluxation of the atlantoaxial joint. It usually affects children and typically presents with torticollis after ear, nose, and throat (ENT) surgery or head and neck infections. In the pediatric literature, there is only a small amount of available data; moreover, no systematic review has been previously done with focus on the pediatric population. We report our experience of two cases, and we provide a systematic review on Grisel's syndrome in children in order to offer a deeper insight about its clinical presentation, its current diagnosis, and principles of treatment. *Case Reports and Review*. We describe two boys of 9 and 8 years old, who developed atlantoaxial subluxation after adenoidectomy. Considering the early diagnosis, a conservative treatment was chosen, with no recurrence and no sequelae at follow-up. We identified 114 case reports, of which 90 describe children, for a total of 171 pediatric patients. Of the 154 cases in which cause was reported, 59.7% presented a head and neck infection and 35.7% had previous head and neck surgery. There is no sex prevalence (49.7% males versus 50.2% females). Mean delay in diagnosis is 33 days. Eight % of the patients had neurological impairment of the 165 cases which mentioned treatment, 96% underwent a conservative treatment, of whom the 8.8% recurred with the need of surgery. As a whole, 12% underwent surgery as a first- or second-line treatment. 3 6% of the patients whose follow-up was reported developed a sequela, minor limitation of neck movement being the most frequent. *Conclusion*. Grisel's syndrome should be suspected in children with painful unresponsive torticollis following ENT procedures or head and neck inflammation. CT scan with 3D reconstruction is the gold standard for diagnosis, allowing the identification of the subluxation and the classification according to the Fielding–Hawkins grading system. Surgical treatment is indicated in case of high-grade instability or failure of conservative treatment. Review of the literature shows how early diagnosis based on clinical and radiological evaluation is crucial in order to avoid surgical treatment and neurologic sequelae.

## 1. Background

Grisel's syndrome (GS) is defined as a rotatory subluxation of the atlantoaxial joint not associated with trauma or bone lesion. It is a rare condition which can be mostly observed in children, though it was first described in adult patients [1–4].

It is usually associated either with infection of the upper airways (pharyngitis, otitis, and mastoiditis) or head and neck regions (lymphadenitis and retropharyngeal abscess), or with ear, nose, and throat (ENT) surgical procedures (tonsillectomy, adenoidectomy, mastoidectomy, tympanoplasty, uvulectomy, or cochlear implantation) [5]. The high predominance of cases in children is probably linked to the combination of various elements: greater ligamentous laxity of the cervical region, hypermobility of C1 on C2 with greater atlas-dens interval, weak cervical muscles, immature bone formation, horizontally oriented facet joints, larger synovial folds in occipito-atlanto-axial joints, adenotonsillar hypertrophy, and a higher rate of upper airways infections [6–9].

The diagnostic workup is based on clinical and radiologic evidence. GS should be suspected in patients presenting with painful torticollis and fever, restricted range of motion, and pain on attempted reduction, in the absence of trauma, but with a history of upper airways or head and neck infections, or ENT surgery [3, 10]. Radiograms and CT scans of the cervical spine are required to confirm the diagnosis and to describe the degree of displacement of the atlantoaxial joint, which enables the classification in the Fielding–Hawkins grading system (Table 1) [11]. MRI can add useful information about abnormalities of soft tissues, lymph nodes, ligaments, and neural structures. It is crucial for the evaluation of the spinal cord when neurologic signs and symptoms are present [5, 7, 12]. There is no gold standard management for this condition, even though there is general consensus on the use of a progressively more invasive treatment according to the Fielding–Hawkins grade and to the delay in diagnosis [5, 8, 13].

We report on two cases and present a systematic review of the literature describing GS in childhood in order to provide an extensive overview of the pathogenic theories, clinical presentation, and diagnostic workup and treatment options of this rare condition.

### 1.1. Case 1

A 9-year-old boy was admitted to the Pediatric Emergency Unit (PEU) for cervical pain, torticollis, and fever (up to 38.5°C) since the previous day. There was no history of trauma, but 2 days earlier, he underwent a surgical adenoidectomy and turbinoplasty, with uneventful postoperative period. At examination, the patient presented torticollis, with pain at every attempted movement of the neck, and pharyngeal hyperemia. Neurologic and systemic physical examinations were normal. Exams showed neutrophilic leukocytosis (14,870/mm^3^) and mild increase of CRP (3.3 mg/dl). Cervical spine X-ray (anteroposterior, lateral, and transoral odontoid projections) was deemed normal. Otorhinolaryngological evaluation reported a normal postoperative situation. The patient was discharged with prescription of ibuprofen, amoxicillin, and the positioning of a soft neck brace.

**Figure 1:**
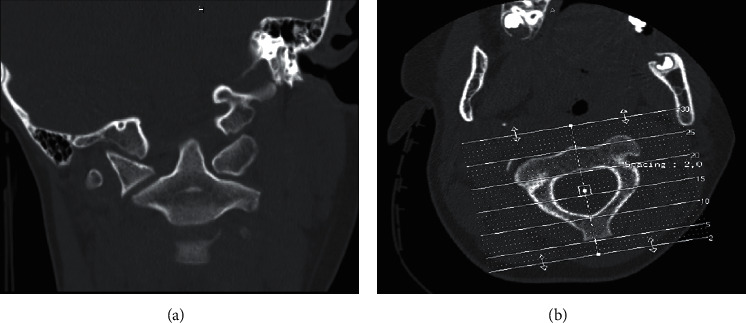
CT scans of our patient 1. (a) The anteroposterior view shows the asymmetry of the lateral masses of the atlas with respect to the axis. (b) The transversal view shows the rotation of the atlas on the axis.

**Figure 2:**
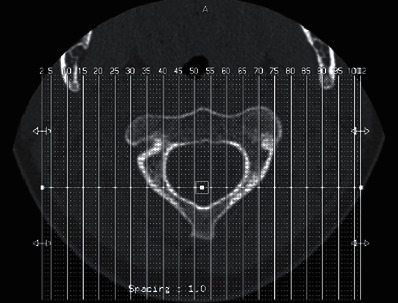
CT scans at follow-up of patient 1 show realignment of the atlas with the axis.

The following day, the patient came back to PEU. He referred worsening of the painful torticollis, with extension of the pain to the dorsal region, and two episodes of vomiting. Physical examination showed torticollis with the head flexed to the right, chin tilted to the left, important spasm of the left sternocleidomastoid muscle, and limited range of motion with severe pain on attempted reduction. Pharyngeal hyperemia persisted, with no signs of retropharyngeal abscess. No lymphadenomegalies, no deficit of the cranial nerves, and no paresthesia were noted. To rule out retropharyngeal abscess and to evaluate the alignment of the cervical spine, we performed cervical MRI scan. The MRI showed presence of C1-C2 rotary subluxation, a fluid collection in the prevertebral space, purulent pharyngeal collection without clear abscess formation, marked edema, and thickening of paravertebral muscles. Fiberoptic laryngoscopy completely excluded retropharyngeal abscess. After neurosurgical consultation, treatment was started with analgesics, antibiotics (ceftriaxone and metronidazole), corticosteroids to reduce edema, the positioning of Philadelphia collar, and bed rest with maximum 30° mobilization of the torso. CT scan with multiplanar reconstructions confirmed the atlantoaxial rotary subluxation, with an increase in the atlanto-dens interval of 6 mm (Figure 1), corresponding to a Type III GS. Manual closed reduction was performed and CT scan showed complete resolution of the subluxation. Philadelphia brace was positioned and maintained for 1 month, and the antibiotic treatment was continued for 13 total days, with no recurrence of the subluxation at the clinical and radiologic follow-up (Figure 2).

### 1.2. Case 2

An 8-year-old boy presented to PEU, reporting a syncopal episode during shower, accompanied by cervical pain and torticollis. Eight days before, he underwent a surgical adenoidectomy, and 6 days before, he had been evaluated for torticollis and discharged with anti-inflammatory (ibuprofen) and analgesic (paracetamol) therapy, without benefit. At physical examination, spasm of the right sternocleidomastoid muscle and cervical stiffness with head tilted to the right were observed. Blood exams and ECG were normal (WBC 11.320/mm^3^, CRP <0.2 mg/dl). Cervical spine CT scan revealed rotatory atlantoaxial subluxation and a hypodense area in the right retropharyngeal tissues. The patient was hospitalized and initially managed with immobilization in bed; therapy with ceftriaxone and ketorolac was started.

The following day, he underwent a manual reduction of the atlantoaxial subluxation under narcosis, and the restoration of a normal articular rapport was confirmed by cervical CT scan. Following the reduction, an Aspen collar was positioned for two weeks. The patient had no recurrence and he did not develop neurological sequelae.

## 2. Methods

### 2.1. Information Sources

In order to achieve a systematic review of the pediatric literature about GS, we performed a search of MEDLINE through the PubMed interface, using the keywords “Grisel Syndrome,” “Grisel's Syndrome,” and “Non-traumatic atlantoaxial subluxation” filtered for age. No filters for time or type of article were used, and last search was performed on 19^th^ April 2020. English and non-English literatures were reviewed, and articles in non-English languages were translated. Moreover, a manual search was conducted through the bibliography of the most relevant articles. More details on the methods are presented in Supplementary File 1.

## 3. Results

### 3.1. Papers Selection

**Figure 3:**
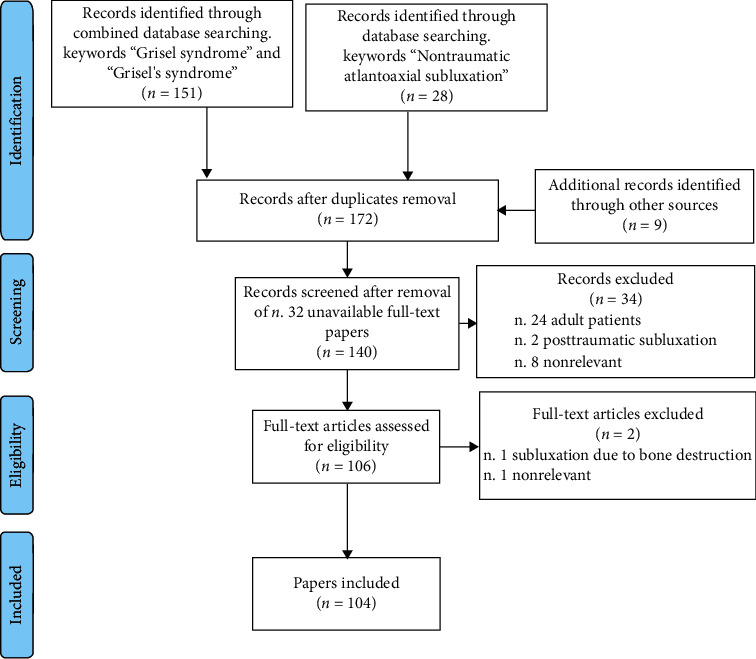
PRISMA flow diagram of papers selection [19].

Overall, 151 papers were identified through the combined search with keywords “Grisel syndrome” and “Grisel's syndrome,” while 28 were obtained from the search with keyword “Nontraumatic atlantoaxial subluxation,” with a total of 179 papers. After eliminating doubles, of the remaining 163 papers, 32 were excluded due to unavailability of full text, and the other 131 studies were evaluated for inclusion with a first screening through the title and abstract. This first screening allowed us to identify and exclude 34 studies, namely, 24 case reports concerning adult patients, 2 case reports describing posttraumatic atlantoaxial subluxation, and 8 nonrelevant papers (2 radiology quizzes, 1 review of surgical procedures, 1 paper describing complications of adenoidectomy without focus on GS, 1 case report describing torticollis in a conversion disorder, and 3 non-GS-related studies). The remaining 97 papers were evaluated through text reading, and 2 papers were excluded, (the first was nonrelevant, while the second described a form of subluxation originated from odontoid bone destruction, which did not appear to share the classic pathogenesis of GS). In conclusion, 95 articles were selected for inclusion in our review, to which 9 articles were added, obtained from manual search through the bibliography of most relevant papers [1, 2, 7, 11, 14–18], with a total of 104 papers included, of which 90 were case reports or case series (Figure 3) [19] (Supplementary File 1 

### 3.2. Synthesis of Results (Table 2)

We identified a total of 171 pediatric patients with GS, aged between 5 months and 14 years (set age limit), with a mean of 7.5 years. Among these, considering the cases in which the possible cause was reported, 92/154 presented an upper respiratory tract or head and neck infection (59.7%). In addition, 1 patient developed GS after bronchitis and 2 patients after meningitis; 1 case was reported in a patient with recurrent esophagitis and 1 in a patient with gastroenteritis. Notably, 2 cases of GS were reported after Kawasaki syndrome. Among the 154 patients, 55 had recent history of ENT or craniofacial surgery (35.7%), of whom the most frequent surgeries were adenotonsillectomy, which represented the 67.2%, mastoidectomy, and cochlear implantation, each representing the 5.4%. There is no sex prevalence (49.7% male versus 50.2% female affected).

Only 4 cases were reported of patients with craniofacial anomalies, which might facilitate the development of GS. This low incidence is highly probably linked to the usual exclusion of such cases during the process of the retrospective review of cases in the single papers. The mean delay in diagnosis is 33 days (from 1 day at least to a maximum of 8 months). The most frequent types were Type I and II of the Fielding–Hawkins classification.

The clinical presentation was usually painful torticollis in the classic cock-robin position, with impossibility of turning the head past the midline, sometimes associated with fever. In 8% (14/171) of the patients, neurological impairment was present at clinical evaluation, including 2 cases of cranial nerve deficit (involving IX, X, and XII nerves, with slurred speech and/or palatal weakness) and 1 case of quadriplegia with respiratory distress in a patient with probable previous atlantoaxial instability. Other forms of neurological deficit included arm, shoulder, or neck muscle weakness, facial neuralgia and radicular pain irradiating down shoulders. Neurological symptoms resolved after treatment of the atlantoaxial subluxation.

Overall, of the 165 cases in which treatment was discussed, 96% of patients (159/165) underwent a conservative treatment, as we considered the combination of all kinds of treatment which did not involve open surgery with arthrodesis or tenotomy, such as medical treatment, positioning of soft or hard collar, use of halo vest, halter traction, and closed manual reposition. Of those patients, the 8.8% (14/159) recurred with the need of surgical arthrodesis or, in one case, of a tenotomy of the sternocleidomastoid muscle. As a whole, 12% (20/165) underwent surgery as a first- (3.6%) or second-line (8.5%) treatment. Only 6 out of the 164 (3.6%) patients whose follow-up was reported developed a permanent impairment after being treated for GS, the most frequent being a minor limitation of the neck movement (4 cases). In addition, one patient developed a slight C1-C2 hypermobility, while another patient, who developed GS after mononucleosis, developed ankyloses between the occiput, C1, and C2.

## 4. Discussion

### 4.1. Pathogenesis

In our review, we observed a higher number of patients who developed GS after upper airways or head and neck infection (57.7%) than after ENT surgery (37.8%). This result supports the findings of Karkos et al., while it contrasts the data *s* of previous papers which deemed ENT surgery to be the most frequent cause [20–22].

Several theories have been put forward to explain the pathophysiology of this syndrome. Grisel firstly thought that torticollis and subluxation were caused by spasm of the paravertebral and cervical muscles, following an infectious process [2]. Other elements that were thought to contribute were hyperemia, which was supposed to cause decalcification of the insertion of the transverse ligament, and regional inflammation, which could lead to pathologic laxity of the atlantoaxial ligaments [3]. This particular theory seems to find its anatomic basis in the studies of Parke et al. [23], who described the possible pathway for spread of inflammation from the pharynx to the atlantoaxial ligaments: a system of pharyngovertebral veins which originate in the postero-superior pharynx, traverse the prevertebral fascia, and drain in the periodontoidal plexus. Anastomoses between pharyngovertebral veins and lymphatic vessels of the periodontoidal plexus would allow this communication, thus enabling the spread of infectious emboli and the development of prevertebral fasciitis, abnormal laxity of atlantoaxial ligaments, and a reactional muscle contraction: It would progressively cause torticollis and eventually subluxation [3, 21, 24]. However, little evidence has been found linking hyperemia, or septic infiltrate, with decalcification or ligamentous laxity [21, 25]. This encouraged Welinder et al. to postulate that distention of atlantoaxial ligaments due to the spasm of irritated cervical muscles, rather than their loosening by the spreading infection, creates the basis for subluxation. In particular, when this spasm occurs in the presence of preexisting laxity of ligaments, subluxation would be facilitated [10, 21]. In this context, Battiata et al. proposed a two-hit hypothesis for the development of GS: The first hit would be the preexisting cervical ligamentous laxity and hypermobility of the atlas-dens joint in children, as seen by the increased atlas-dens interval, which ranges from 2.5 mm to 3 mm in adults, while it can reach 4.5 mm in children. The second hit would be represented by muscle spasm caused by infection. This mechanism would explain why children are more frequently affected by GS, and the association with other conditions characterized by increased ligamentous laxity, such as Down syndrome and Marfan syndrome. Other conditions at risk are Klippel-Feil syndrome, osteogenesis imperfecta, neurofibromatosis, or any syndrome associated with spinal instability, such as juvenile idiopathic arthritis [8, 15, 26]. The association with Kawasaki disease has been reported in some cases, and this lead to the hypothesis of a relationship between atlantoaxial subluxation and autoimmune chronic inflammation [27, 28]. As far as ENT surgery is concerned in the development of GS, the role of excessive passive rotation and hyperextension of the head during the procedures, or even the role of the patient being transferred to hospital bed without use of a roll board, have been pointed out [4]. Another risk factor for the development of GS could be the use of monopolar suction electrocautery during adenotonsillectomies, as it is suggested in the retrospective study by Tschopp et al., which reported an increase of GS cases after the introduction of monopolar suction electrocautery [29, 30]. On the other hand, the study by Henry et al. states that complications of adenotonsillectomy appear to be independent of adenoidectomy technique and cautery use [31, 32].

Overall, despite numerous hypotheses, the pathogenesis of GS has still not been completely understood.

### 4.2. Diagnosis

Diagnosis should be based on a high index of suspicion, in every patient who presents painful torticollis after upper airways or head and neck infection, or after ENT surgery. In fact, GS is a condition which can often go unrecognized, especially when associated with ENT surgery, because of the tendency to treat the symptoms as usual post-tonsillectomy malaise and pain. In our Case 1, the patient underwent a postoperative evaluation which had deemed the situation as a normal postsurgery course.

On physical examination, the patient is unable to rotate the head past the midline, and a distinguishing feature from other causes of torticollis is the spasm of the longer sternocleidomastoid muscle ipsilateral to the tilted chin, as opposed to spasm of the contralateral in other forms. The spinous process of the axis can be palpated in the neck away from the midline toward the affected side (Sudeck's sign); there can be reduction in size of the nasopharynx due to atlas anterior displacement, and the mass effect can also produce a nasal quality of the voice [3, 10, 33].

Early diagnosis, based on clinical and radiological evidence, is crucial in order to avoid the development of neurologic complications due to the spinal cord or radicular compression. According to previous reviews, neurologic sequelae can range from mild paresthesia, local pain, and clonus, to cranial nerve palsies, quadriplegia, acute respiratory failure, and death [4, 12]. In our systematic review, we identified 14 cases with neurological impairment at onset, among which 2 cases were cranial nerve deficit (involving IX, X, and XII nerves, presenting with slurred speech and/or palatal weakness) [34, 35]. A peculiar case was presented by Agarwal et al. [18], who described a patient, with probable previous atlantoaxial instability, who developed quadriplegia with respiratory distress after undergoing adenotonsillectomy. Other forms of neurological deficit reported in literature include clonus, arm, shoulder or neck muscle weakness, hyperreflexia, facial neuralgia, and radicular pain irradiating at shoulders [36–44].

Diagnostic workup requires a thorough neuroradiological examination, including plain X-rays, CT scan, and MRI scan. Radiographs of the upper cervical spine need to be performed in the anteroposterior, lateral, and transoral odontoid projections. Transoral views can provide a good visualization of rotational deformity; anteroposterior views reveal asymmetry between the facet joints, while lateral views show an increased atlantoaxial interval: an increase greater than 4.5 mm is suspect for subluxation [4–6]. However, diagnosis through radiograms may not be very reliable, as on the one hand, it is possible to miss a slight displacement (a plain X-ray deemed as negative does not rule out subluxation), and on the other hand, odontoid asymmetry noted on the transoral view is a common finding and probably a normal variant [17]. In our Case 1, radiographs obtained during the first admission to the emergency department were reported as normal, but it is not sure whether there was actually no subluxation, and it developed subsequently or it was a false negative, given the fact that up to the following day, it had already evolved to a type 3 subluxation.

Therefore, the gold standard for the diagnosis of atlantoaxial rotary subluxation is CT scan with three-dimensional reconstruction, which not only allows the identification of the atlantoaxial subluxation but also the evaluation of the atlanto-dens interval, thus enabling the classification according to the Fielding–Hawkins grading system [8]. MRI is helpful for the evaluation of soft tissues, lymph nodes, ligaments, and neural structures such as spinal cord. Even though MRI does not represent the gold standard for diagnosis, it is often the first radiological examination to be performed when GS is suspected, and there are authors who suggest it could be a reasonable indication to perform MRI first in order to reduce the risk of radiation exposure. CT scan would still be indicated, if MRI reveals subluxation, to classify the atlantoaxial displacement [5]. In our Case 1, MRI was performed first in order to rule out retropharyngeal abscess as a postsurgery complication, and it was then completed by CT scan evaluation; in Case 2, it has never been performed. The role of radiology is also to distinguish GS from other possible differential diagnoses, such as cervical cord or posterior fossa tumors, syringomyelia, cervical malformations, painful adenopathy, retropharyngeal abscess, bone lesions, and traumatic subluxation [14, 26].

### 4.3. Treatment

There are still no clear guidelines for the management of this condition, even though various recommendations have been proposed through the years. The widespread approach is based on the use of increasingly invasive treatment according to the Fielding-Hawkins grade of subluxation (Table 1), which is related to the risk of neurologic complications. In particular, Type I and Type II are usually not associated with neurologic impairment, while Type III and Type IV are associated with up to 15% incidence of neurologic complications [26]. Delay in diagnosis is the other key element to consider when deciding the management of GS, given its direct relationship with the risk of neurologic impairment, failure in conservative treatment, and greater recurrence rate. In the Fielding-Hawkins classification, Type I is characterized by simple rotation, without anterior displacement of the atlas on the axis, or with anterior displacement <3 mm, while Type II is characterized by rotation of the axis associated with anterior displacement >3 mm and <5 mm [11]. Type I and Type II are the most frequent forms of subluxation and are usually treated conservatively, with antibiotic and anti-inflammatory drugs, bed rest, and external immobilization for 4 weeks (soft collar for Type I and hard collar for Type II) [8]. However, treatment needs to be individualized according to the specific patient, and occasionally, Type I and II may need more aggressive management in case of neurological symptoms or failure of treatment. Some authors have proposed to include in the classification a Type 0, for cases of persistent torticollis with the characteristic presentation of GS, but without subluxation at radiologic workup, suggesting this to be the first phase of the step-by-step evolution of the disease [45, 46].

Type III is characterized by rotation with anterior displacement >5 mm, while Type IV consists of rotation of the atlas with posterior displacement and is a very rare condition. Type III and Type IV usually require bed rest with cervical halter traction, followed by a period of neck immobilization with halo vest. Surgical arthrodesis may be necessary in case of failure of the conservative treatment and is often considered the first approach in Type IV cases [4, 5]. Although several cases have been reported having a positive outcome with no lasting deficits after fairly aggressive treatment [13], it has been recently questioned whether it would be possible to obtain equally full recovery with conservative treatment, without having to resort to surgery. In fact, even though surgical arthrodesis is a safe procedure, preventing recurrence and allowing earlier recovery, it inevitably leads to loss of a certain degree of rotational function of the upper cervical spine. In this view, the new technique of closed manual repositioning was proposed. Following the closed reduction maneuver, patients are usually treated with antibiotic and anti-inflammatory medications for at least 1 week and wear external orthosis for a period of 4–6 weeks to prevent recurrence. In particular, the halo vest and a longer immobilization period should be first choice after closed reduction when the delay in diagnosis is >4 weeks. This technique has been proven to allow fast and pain-free reduction, with drastic shortening of the period of hospitalization, and seems to be associated with low recurrence rate. However, its effectiveness has mainly been demonstrated in Type I and Type II subluxations, either as first choice, most of all after delayed diagnosis, or after failure of a first approach with drugs and cervical collar [7, 21, 33, 42, 47–50].

Only few papers have addressed the therapeutic management of Type III, and it remains unclear whether it should receive invasive treatment or not. Spennato and colleagues reported a case of Type III GS, which was successfully treated with surgery after failure of medical treatment [13] while Lee and colleagues directly addressed the issue, of which treatment is the most appropriate for Type III subluxation. They described 6 cases of children with Type III GS, all of which were initially treated conservatively with halter skeletal traction, but in 5 out of 6 cases, surgery was required for recurrence or inefficacy. Lee and colleagues concluded by recommending the use of surgical treatment in Type III atlantoaxial rotational subluxation in patients with a delay in diagnosis greater than 6 weeks [17].

On the other hand, Pilge and colleagues proposed a novel and simplified therapeutic approach, which aims to employ less invasive techniques, irrespectively of the underlying Fielding–Hawkins classification (Type I–III only). They treated 5 patients ranging from Type I to III with manual repositioning under general anesthesia, followed by immobilization of the cervical spine for 4–8 weeks, and obtained good clinical outcome. Overall recovery time and hospital stay were reduced, if compared to the more invasive approach, and it was possible to avoid halo vest positioning and surgery in Type III patients, even in case of delayed diagnosis [48]. Other three cases are reported in the literature of effective manual reduction in a Type III GS [40, 50, 51].

Mahr and colleagues proposed a new treatment algorithm of GS, a “therapeutic crescendo,” which starts with a “wait and see” approach involving analgesic therapy and immobilization for the first 3 days and then manual closed reduction if the symptoms persists, followed by immobilization with the rigid collar. If the subluxation recurs, one attempt of manual closed reduction can be repeated, followed in this case by halo vest immobilization, but in case of further recurring subluxation, open reduction is then proposed [43]. Anania and colleagues have stressed the importance of choosing treatment according to whether the subluxation is recent (<1 month) or inveterate (>1 month) and proposed an algorithm which differs from those employed by PIlge and Mahr. Indeed, they suggest the use of halo vest fixation and reduction with immobilization for 3 months in case of recent subluxation, while halo fixation with reduction followed by surgical arthrodesis is preferred for inveterate subluxation cases [8].

In our case, a 9-year-old patient with Type III subluxation and an 8-year-old patient with an unspecified grade of subluxation have been similarly treated, undergoing manual reposition and subsequent immobilization with Philadelphia collar, with no recurrence at follow-up. Early diagnosis and prompt treatment, (only 5 days after the first symptoms in the first case and 6 days in the second case) allowed to avoid cervical traction, open surgery, and immobilization with the halo vest, which is often burdened by low compliance. Drawing from our Case 1, delay in diagnosis is possibly the key element which should indicate if Type III should be treated conservatively or with more invasive treatment.

### 4.4. Limits

The main limit of this reviewing process lies in the literature being represented by numerous case reports, with only few case series, and even fewer retrospective studies. No randomized controlled trials have been conducted to decide which treatment represents the best option, given the difficulty in summoning a sufficient number of cases and the possible sequelae in case of failure.

## 5. Conclusion

GS should be suspected and investigated in any children with atraumatic painful torticollis, following ENT surgery or upper airways or head and neck infections. Early diagnosis based on clinical and radiological evaluation is crucial for prompt and appropriate management, which is particularly important in order to avoid neurologic sequelae. In our 2 cases, the early diagnosis and treatment allowed to avoid invasive treatment, thus supporting the algorithm proposed by Mahr et al. [43, 48]. Neurosurgical consultation should be obtained, and the choice of treatment should be based on the Fielding–Hawkins grading system. Both the type and the delay in diagnosis are directly related to the risk of neurologic impairment. Subluxation develops progressively and is usually preceded by a period of torticollis and fever. Therefore, it is crucial to employ conservative treatment in this period in order to avoid subsequent development of subluxation [26]. Nevertheless, surgical arthrodesis is a safe and effective tool, which is required in cases of delayed diagnosis (>3 months according to Fielding et al. [11]), in Type IV subluxation and in Type I–III, if neurologic symptoms are present or in case of failure of the conservative treatment. One month after surgery, follow-up clinical examination is needed to evaluate for possible neurologic sequelae.

## Figures and Tables

**Table 1:** Fielding–Hawkins classification.

Type 1	Rotation of the atlas on the axis without displacement, or with anterior displacement ≤3 mm
Type 2	Rotatory fixation with anterior displacement of the atlas 3–5 mm
Type 3	Rotatory fixation with anterior displacement of the atlas ≥5 mm
Type 4	Rotatory fixation with posterior displacement (extremely rare condition)

**Table 2:** Synthesis of results.

Items	Results
Number of patients	171 (49.7% M, 50.2% F)

Age (mean)	7.5 yrs (range 5–14 yrs)

Causes^a^	URTI^b^/head and neck infections = 59.7%
	ENT surgery = 35.7%
	Others = 4.5%^c^

Mean delay in diagnosis	33 days

F–H classification^d^	Type I = 43.6%
	Type II = 35.6%
	Type III = 18.8%
	Type IV = 1.9%

Neurological impairment	8% (14/171 patients)

Treatment	96% conservative
	8.8% recurrence after conservative treatment
	3.6% first-line surgery

Outcome^e^	3.6% permanent impairment reported

^a^Percentage referred to the 154 cases with known cause; ^b^URTI: upper respiratory tract infection; c: 1 bronchitis, 1 gastroenteritis, 1 recurrent esophagitis, 2 Kawasaki syndrome, 2 meningitis; ^d^Percentage referred to the 101 cases with known F–H type; ^e^Percentage referred to the 164 cases with known outcome.

## Data Availability

All data generated or analyzed during this study are included in this article and its supplementary information files. The data supporting this systematic review are from previously reported studies and datasets, which have been cited. The processed data are available in the supplementary file and from the corresponding author upon request.

## Abbreviations

ENT: Ear, nose, and throat
CT: Computed tomography
3D: Three-dimensional
GS: Grisel's syndrome
MRI: Magnetic resonance imaging
PEU: Pediatric Emergency Unit
CRP: C-reactive protein
ECG: Electrocardiography
WBC: White blood cells.

## Supplementary Materials

Supplementary Materials Supplementary File 1: details of methods. Supplementary File 2: reference database. Supplementary File 3: CARE check list.
