# Supplementary material for: Grisel's Syndrome in Children: Two Case Reports and Systematic Review of the Literature
Source: Case Rep Pediatr. 2020 Nov 12;2020:8819758. doi: 10.1155/2020/8819758 (PMC7676959; doi:10.1155/2020/8819758)
Supplement: Supplementary Materials — Supplementary File 1: details of methods. Supplementary File 2: reference database. Supplementary File 3: CARE check list. [file 8819758.f1.zip › Supplementary file 1 Details of methods.docx]

Additional file: 1 Details of methods

Eligibility criteria

Single case reports and case series of pediatric age only were included. Age limit was set at 14 years old, 14-year old patients included. Previous reviews and original articles proposing management algorithms were included, as well as articles describing pathogenic hypotheses, possible roles played by ENT surgery techniques in triggering atlantoaxial subluxation, postoperative complications of ENT surgery with focus on GS and possible neurologic sequelae of this condition. Articles presenting GS variants, with involvement of C2-C3 or C3-C4, were included.

Traumatic forms of atlanto-axial subluxations were excluded, as GS is defined as no traumatic, alongside with forms whose pathogenic mechanisms didn’t match the classic pathogenesis of GS, such as cases where subluxation was triggered by bone destruction. Of the case series which included both traumatic and no traumatic cases, only the no traumatic ones were considered. Surgery techniques’ reviews were excluded.

Papers selection

Two authors separately conducted the selection of the records obtained from the search of MEDLINE through keywords “Grisel Syndrome” / “Grisel’s Syndrome”, and those obtained through keywords “Non-traumatic atlantoaxial subluxation”. Duplicates were removed and the remaining records were screened firstly on the basis of title and abstract, then through full-text reading, according to the eligibility criteria.

Data collection

We performed the extraction of two kinds of data, namely (1) data about the core elements of the cases and (2) data describing the key points of pathogenesis, clinical presentation, diagnosis and management of GS. The items considered during the extraction of the first kind of data were: how many cases were presented in the paper, age and sex of the patients, whether or not an underlying disorder was mentioned (which could possibly represent a risk factor for the development of subluxation), the cause which triggered the subluxation, the presence of neurological impairment at the clinical examination, the delay in diagnosis (we considered days as unit of measure), the Fielding-Hawkins grade, how the case was treated and the outcome, that is whether or not there was recurrence of subluxation with the need for open surgery and whether there were sequelae. Most data were explicitly stated in the papers, while others needed sometimes to be inferred, such as Fielding-Hawkins grade which could be calculated from the CT scan reports, or the delay in diagnosis, which often required to be assumed from the given information, and which we counted using the unit “day”, with the simplification of considering 1 month=30 days. In four cases both ENT surgery and upper respiratory tract infection were present in the patient’s history (i.e.: mastoidectomy for otitis media). In such cases we based the identification of the cause on the temporal relation to the onset of torticollis (i.e.: patient with otitis media and torticollis which undergoes mastoidectomy was considered in the category of GS due to infection).

In four cases we found, through the confrontation of the names of the authors, the same case to be featured in two different papers from the same study group. In those circumstances we decided to include both studies, but the case was counted only once.
