# Supplementary material for: Grisel's Syndrome in Children: Two Case Reports and Systematic Review of the Literature
Source: Case Rep Pediatr. 2020 Nov 12;2020:8819758. doi: 10.1155/2020/8819758 (PMC7676959; doi:10.1155/2020/8819758)
Supplement: Supplementary Materials — Supplementary File 1: details of methods. Supplementary File 2: reference database. Supplementary File 3: CARE check list. [file 8819758.f1.zip › Supplementary file 2.docx]

| **Author** | **Year** | | **N. cases** | **Sex** | **Age (years)** | **Underlying disorders** | **Neurologic impairment** | **Cause** | **F-H Type** | **Delay in diagnosis (days)** | **Treatment** | **Outcome** |
| --- | --- | --- | --- | --- | --- | --- | --- | --- | --- | --- | --- | --- |
| Lucas JT (44) | 1982 | | 1 | F | 9 | - | winging of the right scapula; weakness of abduction of the right arm at the shoulder; hyperactive right knee jerk. | URTI: acute rheumatic fever | - | 120 | Conservative | relapse first, no recurrence after second traction |
| Boiten J (52) | 1986 | | 2 | 1F, 1M | 5,6 | - | - | URTI | - | - | Conservative | no recurrence |
| Sangermani R (53) | 1986 | | 1 | F | 7 | - | - | ENT surgery: AT | - | - | - | - |
| Wilson BC (3) | 1987 | | 1 | M | 6 | - | Meningitis | RP cellulitis following  Meningitis | - | 20 | Conservative | no recurrence |
| Bredenkamp JK (54) | 1990 | | 4 | 2F, 2 M | 5m-11 | - | - | 2 URTI, 1 RPA,1 Sandifer syndrome | - | 1-60 | 3 Conservative/ 1 Surgery | 3 no recurrence/1 frequent relapse  with conservative treatment, no recurrence after surgery |
| Wongsiriamnuey S (55) | 1991 | | 1 | F | 7 | - | - | URTI | I | 3 | Conservative | no recurrence |
| van der Vis-Melsen  MJE (56) | 1992 | | 1 | F | 6 | - | - | URTI | - | 21 | - | - |
| Olivero WC (57) | 1993 | | 1 | F | 3 | - | - | URTI | - | 1 | Spontaneous recover | no recurrence |
| Samuel D (58) | 1995 | | 3 | 2F, 1M | 5-11 | - | - | 2 ENT surgery: M, A /1 URTI | - | - | Conservative | no recurrence |
| Baker LL (59) | 1996 | | 1 | F | 11 | - | - | ENT surgery: AT | IV | 150 | Conservative | decreased range of movement of the neck |
| Welinder NR (10) | 1997 | | 1 | M | 6 | - | - | RPA | II | 60 | Conservative | recurrence, resolution after new skull traction and halo vest |
| Hettiaratchy S (60) | 1998 | | 1 | F | 1 | - | - | URTI | - | 90 | Surgery | no recurrence (reduction neck movement) |
| Lopes DK (61) | 1998 | | 2 | 2F | 9m, 5m | - | - | URTI | C3-C4 | - | Conservative | no recurrence |
| Berry DS (37) | 1999 | | 1 | F | 7 | - | trismus, facial neuralgia | URTI | - | 60 | Conservative | - |
| Garcia-Perez A (62) | 2000 | | 1 | M | 9 | - | - | RPA | - | 2 | Conservative | - |
| Meek MF (63) | 2001 | | 1 | F | 4 | - | - | ENT surgery: P | - | 7 | Conservative | no recurrence |
| Kraft M (45) | 2001 | | 3 | 2F, 1M | 6 | - | - | ENT surgery: A | I/type 0 | 5-17 | Conservative | recurrence with need for stiff collar then traction/no recurrence/no recurrence |
| Holcomb JD (64) | 2001 | | 4 | 2F, 2 M | 7-9 | - | - | 1 URTI + RPA/3 ENT surgery: 1 lipoma removal, 1 Sarcoma removal, 1 TM | - | 1 | 3 Conservative/ 1 Surgery | no recurrence |
| Martinez-Lage JF (36) | 2001 | | 1 | F | 8 | - | 1 with radicular pain irradiating down both shoulders | URTI | - | - | Conservative | no recurrence |
| Gourin CG (38) | 2002 | | 1 | M | 7 | - | 2 beat clonus bilateral | URTI, RP cellulitis, osteomyelitis | II | 14 | Conservative | no recurrence |
| Okada Y (65) | 2002 | | 1 | F | 7 | - | - | Mumps | - | 5 | Conservative | no recurrence |
| Mezue WC (66) | 2002 | | 2 | M | 8 | - | - | URTI | - | - | Conservative | no recurrence |
| Kasten P (67) | 2002 | | 1 | M | 6 | - | - | Lymphadenitis | II | 30 | Conservative | no recurrence |
| Lehtinen P (16) | 2002 | | 1 | F | 8 | - | - | Lymphadenitis | - | 7 | Conservative | no recurrence |
| Lee SC (17) | 2002 | | 6 | 3F, 3 M | 7-12 | - | - | 4 URTI/2 not known | III | 42-150 | 3 Conservative/ 3 Surgery | no recurrence/recurrence with need of C1-C2 artrhodesis/ no recurrence after surgery/recurrence with need of surgery/recurrence with need of surgery/no recurrence after surgery |
| Fernandez-Cornejo VJ (68) | 2003 | | 4 | 1F, 3M | 4-13 | - | - | URTI | II/I/I/I | 1-210 | Conservative | mild restriction of movement/no recurrence/no recurrence/no recurrence |
| Martinez-Lage JF (69) | 2003 | | 1 | M | 5 | - | - | URTI | C2-C3 | - | Conservative | no recurrence |
| Yu KK (6) | 2003 | | 1 | M | 13 | - | - | ENT surgery: AT | - | 2 | Conservative | no recurrence |
| Hirth K (70) | 2003 | | 2 | 2F | 3,7 | - | - | ENT surgery: T/ functional neck dissection | - | 14-180 | Conservative | initial movement reduction, treated with physiotherapy/mild movement reduction, then resolution |
| Feldmann H (71) | 2003 | | 2 | 2F | 11,6 | - | - | ENT surgery: T/AT | - | 12-23 | Conservative | no recurrence/initial recurrence with rotation, no subluxation treated with physiotherapy |
| Wurm G (72) | 2004 | | 1 | M | 3 | - | - | URTI | - | 5 | Conservative | slight hypermobility C1-C2, no recurrence |
| Battiata AP (25) | 2004 | | 1 | M | 11 | - | - | ENT surgery: T | II | - | Conservative | no recurrence |
| Haidar S (34) | 2004 | | 1 | M | 14 | - | IX,X, XII palsy on the right, X palsy on the left | Mononucleosis | - | 150 | - | Occipital-C1-C2 ankylosis |
| Harth M (73) | 2004 | | 1 | M | 11 | - | - | URTI | I | - | Conservative | - |
| Isern AE (74) | 2004 | | 2 | 1F, 1M | 6,8 | - | - | ENT surgery: P | - | 42 | Conservative | Recurrence with need for surgery/no recurrence |
| Galer C (75) | 2005 | | 1 | F | 3 | - | - | URTI | - | 2 | Conservative | no recurrence |
| Henry LR (31) | 2005 | | 1 | - | - | - | - | ENT surgery: A | I | - | - | no recurrence |
| Park SW (76) | 2005 | | 1 | F | 9 | - | - | URTI | II | 90 | Conservative | no recurrence |
| Bocciolini C (4) | 2005 | | 1 | M | 8 | - | - | ENT surgery: A | II | - | Conservative | no recurrence |
| Wieringa JW (77) | 2007 | | 1 | M | 2 | - | meningitis | Meningitis | - | - | Conservative | no recurrence |
| Harma A (78) | 2008 | | 1 | F | 4 | - | - | URTI | II | 2 | Conservative | no recurrence |
| Cekinmez M (79) | 2009 | | 2 | 2M | 8 | - | - | URTI | - | - | Conservative | no recurrence |
| Deichmueller CM (22) | 2010 | | 12 | 6F, 6M | 4-11 | - | - | 8 ENT surgery: 5 AT, 1 T, 1 Tympanoplasty, 1 neck dissection / 3 URTI, 1 G | - | 1-180 | Conservative | no recurrence |
| Pilge H (33) | 2011 | | 1 | F | 11 | - | - | ENT surgery: CI | - | 60 | Conservative | no recurrence |
| Ortega-Evangelio G (26) | 2011 | | 1 | F | 8 | - | - | URTI | I | 8 | Conservative | no recurrence |
| Dagtekin A (80) | 2011 | | 2 | 1F, 1M | 8,14 | - | - | ENT surgery: A/URTI | II/I | 3/- | Conservative | no recurrence |
| Durst F (81) | 2012 | | 1 | F | 9 | - | - | ENT surgery: O | III | 7 | Conservative | no recurrence |
| Nozaki F (27) | 2012 | | 1 | F | 5 | - | - | Kawasaki disease | I | 14 | Conservative | no recurrence |
| Sia KJ (82) | 2012 | | 1 | M | 7 | - | - | ENT surgery: T | I | 4 | Conservative | no recurrence |
| Salpietro V (39) | 2012 | | 1 | F | 7 | - | decreased muscular tone in the shoulder and right neck muscles | URTI | - | - | Conservative | no recurrence |
| Coca-Pélaz A (83) | 2013 | | 1 | M | 3 | - | - | URTI | - | - | Conservative + SCM tenotomy | no recurrence |
| Di Cola F (84) | 2013 | | 1 | M | 6 | - | - | URTI | 0 | 2 | Conservative | no recurrence |
| Pilge H (48) | 2013 | | 5 | 3F, 2M | 8-13 | - | - | ENT surgery: Ch, M, T, CI, O | III/I/II/II/III | 3-60 | Conservative | no recurrence |
| Tweel BC (35) | 2013 | | 1 | F | 8 | - | Palatal weakness, hypernasal speech and difficulty swallowing, likely an isolated deficit of a branch of cranial nerve X | - | I | - | Conservative | no recurrence |
| Wood AJ (28) | 2013 | | 1 | F | 8 | - | - | Kawasaki disease | - | 11 | Conservative | no recurrence |
| Park SH (85) | 2013 | | 1 | M | 9 | - | - | Lymphadenitis | II | - | Conservative | no recurrence |
| Agarwal J (18) | 2013 | | 1 | M | 7 | Retrospectively: pre-existent atlantoaxial instability | Quadriplegia+ acute respiratory distress with cord compression | ENT surgery:AT | - | 1 | Surgery | sleep-induced hypoventilation and hypercapnia |
| Ortiz GL (42) | 2013 | | 3 | 2F, 1M | 7-12 | - | 1 right hemiparesis, right hyperrilfexia, increased right pinprick | 1 ENT surgery: T /2 URTI | II/III/II | -/90/150 | 2 Conservative, 1 Surgery | no recurrence/minor limitation/initial recurrence with surgery need |
| Sogut O (86) | 2014 | | 1 | F | 7 | - | - | URTI | II | 5 | Conservative | no recurrence |
| Bucak A (87) | 2014 | | 1 | M | 8 | - | - | ENT surgery: AT | I | - | Conservative | no recurrence |
| Viscone A (51) | 2014 | | 1 | F | 5 | - | - | URTI | III | 14 | Conservative | no recurrence |
| Barcelos AC (88) | 2014 | | 1 | M | 7 | - | - | URTI | I | 1 | Conservative | no recurrence |
| Akbay A (7) | 2014 | | 1 | - | - | - | - | URTI | - | - | Conservative (Not known if surgery) | - |
| Wang JC (89) | 2014 | | 1 | M | 12 | - | - | ENT surgery: Microtia reconstruction | - | - | Conservative | no recurrence |
| Pavlidis E (90) | 2015 | | 1 | M | 7 | - | - | ENT surgery: AT | I | 3 | Conservative | no recurrence |
| Kourelis K (91) | 2015 | | 1 | F | 9 | Crouzon syndrome | - | ENT surgery: AT | - | - | Conservative | no recurrence |
| Spennato P (13) | 2015 | | 1 | M | 6 | - | - | ENT surgery: A | III | 60 | Conservative then Surgery | recurrence, then surgery C1-C3 fusion |
| Elyajouri A (92) | 2015 | | 1 | F | 8m | - | - | ENT surgery: uvulectomy |  | 1 | Conservative | no recurrence |
| Martins J (47) | 2015 | | 1 | M | 4 | - | - | URTI | II | 6 | Conservative | no recurrence |
| Reichman EF (93) | 2015 | | 1 | M | 4 | - | - | ENT surgery: A | I | 30 | Conservative | no recurrence |
| Allegrini D (94) | 2016 | | 1 | M | 1 | - | - | URTI | - | 5 | Conservative | no recurrence |
| Nakashima T (95) | 2016 | | 1 | F | 7 | - | - | ENT surgery: CI | I | 4 | Conservative | no recurrence |
| Ismi O (96) | 2016 | | 1 | M | 8 | - | - | URTI | I | - | Conservative | no recurrence |
| Das S (97) | 2016 | | 5 | 1F, 4M | 6-12 | - | - | URTI | II/II/II/I/II | 4-10 | Conservative | 4 no recurrence/1 not known |
| Morales LC (41) | 2016 | | 1 | F | 5 | - | electric pain behind right ear | Bronchitis | II | 15 | Conservative then Surgery | non responsive to conservative treatment, no recurrence after surgery |
| Ahn AR (98) | 2017 | | 1 | F | 6 | - | - | Lympahdenitis | I | 1 | Conservative | no recurrence |
| Aladag Ciftedimir N (99) | 2017 | | 2 | 2M | 7,7 | - | - | URTI/Lymphadenitis | I | 2-30 | Conservative | no recurrence |
| Miller BJ (50) | 2018 | | 1 | M | 11 | - | - | ENT surgery: AT | III | 7 | Conservative | no recurrence |
| Stilwell PA (49) | 2018 | | 1 | F | 7 | - | - | URTI | - | - | Conservative | no recurrence |
| Fath L (24) | 2018 | | 2 | 1F, 1M | 10 | - | - | URTI | II | 180-240 | 2 Conservative then Surgery | no recurrence |
| Falsaperla R (100) | 2018 | | 1 | F | 8 | - | - | URTI | - | 7 | Conservative | no recurrence |
| Ozalp H (40) | 2018 | | 16 | 9 M, 7F | 1-14 | - | 1 neurologic deficit | 3 ENT surgery: T, T, A/ 13 URTI | 6I, 3II/3II, 1III, 3I | 1-60 | Conservative | no recurrence |
| Khodabandeh M (101) | 2018 | | 1 | M | 9 | - | - | URTI | - | 16 | Conservative | no recurrence |
| Iaccarino C (5) | 2019 | | 4 | 1F, 4M | 1-7 | - | - | 1 Ent surgery: T/3 URTI | I/I/II/II | 7-90 | Conservative | no recurrence |
| Mahr D (43) | 2019 | | 13 | 7F, 6M | 8,5  (+- 3,8) | - | 12-year-old girl with impairment of muscle strength of the left arm of 4/5 and numbness around the left occiput | ENT surgery/URTI, number non specified | 7I/4II/1III/1IV | - | 7 Conservative/  5 Surgery | 1 recurrence, repeated reduction, 1 surgery after recurrence |
| Anania P (8) | 2019 | | 5 | 4F, 1M | 7-11 | - | - | 4 URTI/1 not known | II/I/II/II/I | 20-180 | 2 Conservative/  3 Conservative then Surgery | no recurrence, one initially complicated |
| Park J (102) | 2019 | | 1 | M | 6 | - | - | ENT surgery: AT | III | 7 | Conservative | no recurrence |
| Cucuzza ME (103) | 2020 | | 1 | F | 6 | - | - | Rheumatic carditis in streptococcal infection | III | - | Conservative | no recurrence |
| Patel V (104) | 2020 | | 3 | 2F, 1M | 6-9 | Crouzon/  Saethre-Chotzen syndrome/  hemifacial mycrosomia | - | Craniofacial surgery | - | 1/2/- | Conservative | no recurrence |
| Bilgin E (105) | 2020 | | 1 | F | 6 | - | - | URTI | I | 7 | Conservative | no recurrence |
|  | | URTI= upper respiratory tract infection, AT= adenotonsillectomy, T=tonsillectomy, A=adenoidectomy, M= mastoidectomy, RP= retropharyngeal, RPA retropharyngeal abscess, G=gastroenteritis, CI=cochlear implant, O=otoplasty, Ch=cholesteatoma, P=pharyngoplasty, TM=timpanomastoidectomy | | | | | | | | | | |
