# Supplementary material for: Grisel's Syndrome in Children: Two Case Reports and Systematic Review of the Literature
Source: Case Rep Pediatr. 2020 Nov 12;2020:8819758. doi: 10.1155/2020/8819758 (PMC7676959; doi:10.1155/2020/8819758)
Supplement: Supplementary Materials — Supplementary File 1: details of methods. Supplementary File 2: reference database. Supplementary File 3: CARE check list. [file 8819758.f1.zip › Supplementary file 3 CARE-checklist-English-2013.docx]

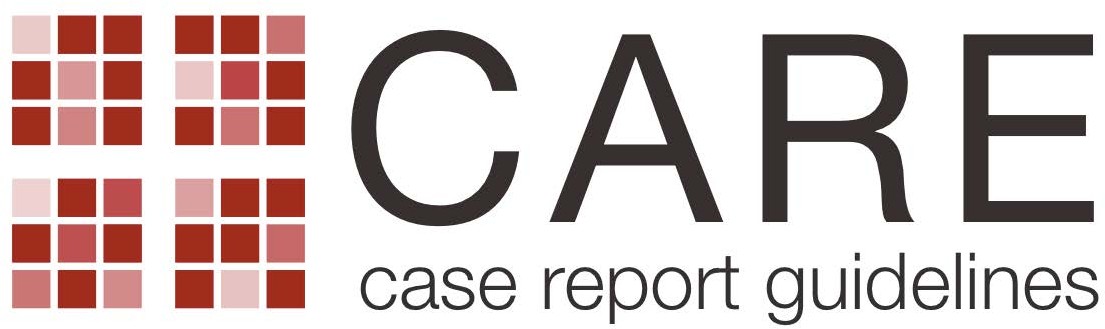
**CARE Checklist of information to include when writing a case report
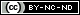
**

**Topic Item Checklist item description Reported on Line**

**itle 1** The diagnosis or intervention of primary focus followed by the words “case report” 1

**Key Words 2** 2 to 5 key words that identify diagnoses or interventions in this case report, including "case report" 32

# Abstract

**(no references)**

**3a** Introduction: What is unique about this case and what does it add to the scientific literature? 28-30

**3b** Main symptoms and/or important clinical findings 12-13

**3c** The main diagnoses, therapeutic interventions, and outcomes 13-14

**3d** Conclusion—What is the main “take-away” lesson(s) from this case? 24-25, 28-30

**Introduction 4** One or two paragraphs summarizing why this case is unique (**may include references**) NA

**Patient Information 5a** De-identified patient specific information 62, 91

**5b** Primary concerns and symptoms of the patient 62-63, 72-73, 91-92

**5c** Medical, family, and psycho-social history including relevant genetic information ………………………..NA___________________

**5d** Relevant past interventions with outcomes …………63-64, 92-94___________

# Clinical Findings

**Timeline**

**Diagnostic Assessment**

**Therapeutic Intervention**

**Follow-up and Outcomes**

1. Describe significant physical examination (PE) and important clinical findings 65-66, 73-77, 94-95
2. Historical and current information from this episode of care organized as a timeline NA

**8a** Diagnostic testing (such as PE, laboratory testing, imaging, surveys). 67-69, 77-86, 96-98

**8b** Diagnostic challenges (such as access to testing, financial, or cultural) NA

**8c** Diagnosis (including other diagnoses considered) 77-82, 84-86, 96-98

**8d** Prognosis (such as staging in oncology) where applicable 86

**9a** Types of therapeutic intervention (such as pharmacologic, surgical, preventive, self-care) 69-71, 82-84, 86-89, 93-94, 98-102

**9b** Administration of therapeutic intervention (such as dosage, strength, duration) 69-71, 82-84, 86-89, 93- 94, 98-102

**9c** Changes in therapeutic intervention (with rationale) 79, 82-84, 97, 98-102

**10a** Clinician and patient-assessed outcomes (if available) 89, 102-103

**10b** Important follow-up diagnostic and other test results 89, 102-103

**10c** Intervention adherence and tolerability (How was this assessed?) NA

**10d** Adverse and unanticipated events NA

**Discussion 11a** A scientific discussion of the strengths AND limitations associated with this case report 244-248, 257-260, 330-337

**11b** Discussion of the relevant medical literature **with references** 168-337

**11c** The scientific rationale for any conclusions (including assessment of possible causes) 311-324

**11d** The primary “take-away” lessons of this case report (without references) in a one paragraph conclusion 345-352

**Patient Perspective 12** The patient should share their perspective in one to two paragraphs on the treatment(s) they received NA

**Informed Consent 13** Did the patient give informed consent? Please provide if requested . . . . . . . . . . . . . . . . . . . . . . . . . . . . . . . . . . . . . . **Yes x No**
